# Environmental Economics for Environmental Protection

**DOI:** 10.1100/tsw.2002.289

**Published:** 2002-05-10

**Authors:** Ekko van Ierland, Corjan Brink, Leen Hordijk, Carolien Kroeze

**Affiliations:** Environmental Economics and Natural Resources Group, Wageningen University, Hollandseweg 1, 6706 KN Wageningen, The Netherlands; Environmental Systems Analysis Group, Wageningen University, Diedenweg 18, 6703 GW Wageningen, The Netherlands

**Keywords:** environmental policy interrelations, cost-effective abatement, acidification, global warming, European agriculture, free-riding, international environmental agreements

## Abstract

Environmental economics deals with the optimal allocation of production factors and correcting market failure in protecting the environment. Market failure occurs because of externalities, common property resources, and public goods. Environmental policy instruments include direct regulation, taxes/subsidies, tradable permits, deposit systems, voluntary agreements, and persuasion.

Environmental policies usually focus on one pollutant or environmental issue but may have substantial impacts on other emissions and environmental problems. Neglecting these impacts will result in suboptimal policies. We present an integrated optimisation model for determining cost-effective strategies to simultaneously reduce emissions of several pollutants from several sources, allowing for interrelations between sources and abatement options. Our integrated approach in regard to acidifying compounds and greenhouse gases will be able to provide cost-effective policy options that will result in lower overall abatement costs.

This paper shows that efficient emission reduction can be calculated, but we argue that, for transboundary air pollution and climate change, it is difficult to implement the socially optimal solution because strong incentives exist for “free-riding”. In order to implement efficient policies, international environmental agree-ments like the Gothenburg or the Kyoto Protocol are necessary to establish stable coalitions. The stability of these agreements depends on the distribution of costs and benefits over countries and on the redistribution of the gains of cooperation.

## INTRODUCTION

Environmental pollution occurs worldwide at a large scale. The main economic reasons for the occurrence of environmental pollution are the focus on economic growth and the opportunity costs of environmental protection. More important, however, are the *failure* of the market to protect the environment and the *failure* of governments to protect the environment in an efficient and effective manner.

The market, highly appreciated for its capability to provide private goods and services in a very efficient way, fails where environmental protection is concerned. The main reasons for its failure are (1) the existence of externalities, (2) the public goods character of the environment, and (3) the common property resource aspects of natural resources, like water, fish, or some stocks of fossil energy resources. Governments fail to protect the environment because of priorities for economic growth in its traditional meaning or because the political support for environmental protection is still insufficient. The failure of the market for environmental protection can be easily shown and hardly needs detailed explanation. *Externalities* occur in many situations, for example, in the case of air pollution. The emissions of one individual have a negative impact on the options of other producers or consumers, without any compensation through the market. This implies that inadequate incentives are provided to producers or consumers to reduce these negative impacts on others. In the 1960s and 1970s, this led to tremendous air pollution problems in Europe and the U.S., calling for immediate environmental policies to reduce air pollution. Similar examples of externalities are water pollution or excessive waste production.

The environment is a *public good* if it is impossible to exclude individuals from the use of the environment and if there exists no rivalry in consumption: if one individual uses the environmental amenity, it does not reduce the options of others to enjoy it. A typical example is the beauty of nature or landscapes. Everyone is enjoying it, and it is impossible to exclude people from enjoying it, and, usually, the use by one individual does not reduce the possibilities of others using it.

Finally, *common property resources* exist, for example, in the case of fisheries. With common property resources, it essentially is possible to exclude people from the right of exploitation of the resources, but property rights are not individually defined. In this case, open access prevails, and it can easily and clearly be illustrated that this leads to overexploitation of the renewable natural resource — for example, ocean fisheries. All individual fishermen have incentives to harvest as much as possible, neglecting the optimal fish stock necessary for regeneration.

An urgent question for policymakers in almost all countries of the world is how to design and introduce environmental policies and at the same time promote further economic development and create sufficient employment opportunities. The failure of the market must be corrected by environmental policy instruments and by the creation of institutions that consider the environmental issues at stake, that provide scientific analysis of the best options to promote sustainable development, and that implement the policies.

For many industrialised countries, international environmental agreements have resulted in commitments to reduce atmospheric emissions of several pollutants. Examples are the "Gothenburg Protocol", aiming at the control of acidification, eutrophication, and ground-level ozone concentrations in Europe[1], and the "Kyoto Protocol", aiming at reductions in greenhouse gas emissions in industrialised countries all over the world[2]. There may be important interrelations between different environmental issues[3]. First, one pollutant may have an impact on several environmental problems. Second, emissions of pollutants may originate from the same human activities (e.g., fossil fuel consumption). Third, technologies for abatement of one pollutant may also affect emissions of other pollutants, either beneficially or adversely. Finally, environmental effects may influence each other. To our knowledge, there are no studies that take into account all of these interrelations. Our study presents a model for the cost-effectiveness analysis of emission-reduction strategies and is designed to include these interrelations. For simultaneously imposed limits on emissions of several pollutants, the model determines which abatement technologies have to be applied to meet these limits at least cost. In a separate paper[4], we used the model in a case study to analyse interrelations between emission-reduction strategies for ammonia ($NH_3$), nitrous oxide ($N_2O$), and methane ($CH_4$) in the agricultural sector in Europe.

The efficient solution for reducing environmental impacts is easy to identify, but its implementation is difficult because strong incentives to “free ride” exist for individual countries. The payoff for free-riding is high, because the countries in the international environmental agreement will pay the costs of the implementation of the policies, while free-riders enjoy the benefits without incurring costs. These incentives for free-riding exist in the problems of both acidification and climate change.

The structure of this paper is as follows: the next section gives the background on the interactions between abatement options and environmental problems. The following section describes the theoretical model developed to analyse cost-effective emission-reduction strategies. The fourth section gives a discussion of environmental policy instruments that may be applied to reach the cost-effective emissions reductions in practice. The last section presents conclusions.

## BACKGROUND

In the past decades, environmental policies have evolved from a uniform reduction percentage for single pollutants to an effect-based approach taking into account several pollutants and several pollution effects. Several studies deal with the linkages between local and regional air pollution problems[4,5]. The Gothenburg Protocol[1] was the first protocol to include several pollutants and several environmental effects in Europe. The RAINS model[i] was used to analyse cost-effective strategies for the reduction of these environmental effects[6].

Global warming will be another important issue in environmental policy making in Europe in the coming years. Policies with respect to global warming mainly focus on carbon dioxide ($CO_2$) but also take into account other greenhouse gases. Interactions between policies to reduce greenhouse gas emissions and policies for other environmental issues (like acidification and tropospheric ozone) are, however, often neglected.

An increasing number of studies pay attention to interactions between abatements of emissions of various compounds, such as greenhouse gases and air pollutants[ii]. These can be subdivided in three groups[7]: (1) literature that primarily looks at reductions in emissions of greenhouse gases and recognises that there may be effects on emissions of air pollutants[8,9], (2) literature that primarily looks at reductions in emissions of air pollutants and recognises that there may be effects on greenhouse gas emissions[10], and (3) literature that looks at the combination of reductions in emissions of greenhouse gases and air pollutants from an integrated perspective (this study).

Examples of interactions mentioned in the literature can be categorised according to the four interrelations described earlier. *First*, emissions may have an effect on global temperature as well as on other environmental problems. For instance, (a) sulphur dioxide ($SO_2$) emissions result in acid deposition and also increased concentrations of sulphate aerosols, which may mask global warming by scattering solar radiation and modifying clouds and their properties[11]; or (b) nitrogen oxides ($NO_x$) emissions contribute to the formation of tropospheric ozone, which has a negative impact on human health and crop production and also contributes to global warming[12]. *Second*, the various greenhouse gases and air pollutants are to a large extent emitted by the same sources. For instance, (a) fossil fuel use results in emissions of the greenhouse gas $CO_2$ and the air pollutants $SO_2$ and $NO_x$[13,14,15]; or (b) agriculture is a source of the greenhouse gases $N_2O$ and $CH_4$ and the acidifying compound $NH_3$[10]. *Third*, technical measures to reduce emissions of air pollutants (like $SO_2$ or $NO_x$) may have an impact on emissions of greenhouse gases and vice versa. For instance, (a) use of three-way catalytic converters in cars reduces emissions of $NO_x$ but results in an increase in emissions of $N_2O$[16,17]; (b) scrubbers installed in power plants to reduce $SO_2$ emissions may increase $CO_2$ emissions through increased coal use[18]; or (c) several measures to reduce $NH_3$ emissions in agriculture have an effect on $CH_4$ and $N_2O$ emissions[19]. *Fourth*, global warming and air pollution may have an effect on each other. For instance, (a) changes in temperature and precipitation affect the rate of acidification, the distribution of air pollutants through the atmosphere, and the sensitivity of ecosystems for acidification and eutrophication[18]; or (b) ground-level ozone production may increase with rising temperatures[20].

To our knowledge, no model exists for analysing cost-effectiveness of abatement strategies for air pollutants and greenhouse gases considering the interrelations mentioned above.

## THEORETICAL MODEL

In this section we present a comparative static optimisation model to determine cost-effective policies for several environmental targets simultaneously, considering interrelations between these policies. The emissions of pollutants are the result of economic activities, which are exogenous to the model. Emissions can be reduced by several abatement options, for which costs and effects on emissions are known. The model is formulated as a stepwise linear program. The linear approach to calculate emissions, costs, and effects of abatement makes it possible to include a large number of sources, pollutants, and abatement options.

For simplicity, we first describe a model for only one pollutant (i.e., ignoring interactions between abatements of different pollutants). Subsequently, the model is extended to include several pollutants and interactions between abatements of these pollutants.

### Single Pollutant, No Interactions

The model minimises total cost of abatement that can be implemented at the various sources to achieve a given constraint on the emission level of the pollutant. The model is described by Eqs. 1 to 6:

$$\min \sum_{k \in K} C_k \left( v_k \right) \text{ with } v_k \text{ a vector with elements } a_{k,n},\, n \in N \qquad (1)$$

subject to

$$C_k \left( v_k \right) = \sum_{n \in N} a_{k,n} \cdot \gamma_{k,n} \cdot \bar{X}_k \quad \forall\, k \in K \qquad (2)$$

$$e_k = \varepsilon_k \cdot \bar{X}_k \cdot \left( 1 - \sum_{n \in N} a_{k,n} \cdot \rho_{k,n} \right) \quad \forall\, k \in K \qquad (3)$$

$$\sum_{k \in K} e_k \leq \bar{E} \qquad (4)$$

$$\sum_{n \in N} a_{k,n} \leq 1 \quad \forall\, k \in K \qquad (5)$$

$$a_{k,n} \geq 0 \quad \forall\, k \in K,\; n \in N \qquad (6)$$

Eq. 1 is the objective function, which minimises the sum of the annual abatement costs $C_k$ over the sources $k \in K$. The decision variables in the model are the application rates $a_{k,n}$ for abatement options $n \in N$ at source $k$ (with $N$ the set of abatement options available). For each source $k$, application rates $a_{k,n}$ for all $n \in N$ are included in the vector $v_k$. Furthermore, abatement costs at source $k$ depend on the (exogenous) activity level $\bar{X}_k$ and the per unit cost of abatement options $\gamma_{k,n}$ for all $n \in N$ (Eq. 2). Annual emissions from source $k$, $e_k$, depend on activity level $\bar{X}_k$, emission factor $\varepsilon_k$, application rate $a_{k,n}$, and effectiveness $\rho_{k,n}$ of the abatement options applied (Eq. 3). Total emissions are constrained in Eq. 4. The application rate $a_{k,n}$ is constrained in Eqs. 5 and 6.

The set $K$ of emission sources in the model may consist of various types of economic activities. The distinction between sources is based on differences in the effect on emissions. The level of economic activity per year at source $k$ is exogenous to the model and indicated by $\bar{X}_k$. A linear relation was assumed between economic activities and unabated emissions at each source, represented by emission factor $\varepsilon_k$.

Total annual emissions cannot exceed level $\bar{E}$ (Eq. 4). To meet this restriction, emissions from each source can be reduced by several abatement options included in set $N$[iii]. Application rate $a_{k,n}$ indicates the fraction of economic activity at source $k$ to which abatement option $n$ is applied (Appendix) and ranges from zero (no application) to one (full application).

The model contains a set of abatement options ($N$), which includes both single abatement technologies and possible combinations of these technologies. Combinations of abatement technologies are included in set $N$ as a new abatement option to avoid nonconvexities and, as a consequence, multiple local optima (Appendix). It is possible to apply several abatement options at the same time to different fractions of an economic activity (Eq. 5)[iv].

The effect of abatement option $n$ on emissions from source $k$ is represented by $\rho_{k,n}$, which indicates the emission reduction as fraction of unabated emissions. The total reduction in emissions by abatement options $n$ is $\varepsilon_k \cdot \bar{X}_k \cdot a_{k,n} \cdot \rho_{k,n}$.

The cost of an abatement option is linearly related to the economic activity[v] level and the application rate. Cost per unit of activity at full application, $\gamma_{k,n}$, may differ between sources. Total cost of abatement at source $k$ is the sum of the costs for the various abatement options (Eq. 2). Overall abatement costs are the sum of costs at the various sources (Eq. 1). Only direct costs of applying abatement technologies (annual investment, operating, and maintenance costs) are included. Secondary impacts on the economy as a whole—for example, as a result of price changes—were not considered.

Abatement cost curves can be constructed to compare abatement costs at various sources[vi]. These cost curves indicate, for emission-reduction levels ranging from zero up to the technically feasible limit, least cost abatement strategies by ranking abatement options according to their increasing marginal costs. To determine marginal cost of abatement options, the cost of abatement option $n$ per unit of emissions abated ($\xi_{k,n}$) is calculated:

$$\xi_{k,n} = \frac{\gamma_{k,n}}{\varepsilon_k \rho_{k,n}} \tag{7}$$

Next, for each source $k$, abatement options are sorted according to the increasing removal efficiency $\rho_{k,n}$. Options that have a lower efficiency ($\rho_{k,n}$) and higher costs per ton reduced ($\xi_{k,n}$) are excluded from the analysis because they will not be applied in a cost-effective solution. For the remaining options, marginal reduction costs are determined [the position of abatement options in the ordered set for source $k$ is indicated by $d(k)$]:

$$\mu_{k,d(k)} = \frac{\xi_{k,d(k)}\ \rho_{k,d(k)} - \xi_{k,d(k)-1}\ \rho_{k,d(k)-1}}{\rho_{k,d(k)} - \rho_{k,d(k)-1}} \tag{8}$$

with $\mu_{k,d(k)}$ the marginal cost of abatement option at position $d(k)$, i.e., the cost of reducing an additional unit of emissions by abatement option at position $d(k)$ instead of the option at position $d(k)$-1 (the next less effective option at the source)[vii]. Abatement options that have a higher marginal cost and a lower efficiency are eliminated, after which marginal costs are determined once more. The remaining options are sorted according to increasing marginal cost of abatement, resulting in abatement cost curves for the various sources. These abatement cost curves are piecewise linear, with kinks indicating changes in marginal abatement cost. A change in marginal abatement cost occurs when abatement option at position $d(k)$ is fully applied, and higher reductions can only be obtained by applying abatement option at position $d(k)$+1 instead, which is more effective, but at higher marginal costs[viii].

To construct an abatement cost curve for the economy as a whole, cost curves of individual sources are integrated into one cost curve by ranking abatement options that can be applied at the various sources according to their marginal abatement costs. For each option, the emission reduction is calculated:

$$r_{k,d(k)} = \bar{X}_k \cdot \varepsilon_k \cdot \left(\rho_{k,d(k)} - \rho_{k,d(k)-1}\right) \tag{9}$$

with $r_{k,d(k)}$ the additional amount of emissions reduced after implementation of abatement option at position $d(k)$ instead of option at position $d(k)$-1[ix].

## Several Pollutants and Interactions

The model described above allows for determining cost-effective abatement strategies for a single pollutant, based on the assumption that this is the only pollutant for which an emission-reduction target exists. The model is extended to include several pollutants $p$ in a set $P$. For each source, emission factors are distinguished for the various pollutants ($\varepsilon_{k,p}$). Targets can be specified for emissions of all $p \in P$ ($\bar{E}_p$).

For multiple emission-reduction targets, overall cost-effectiveness can be obtained by determining cost-effective abatement strategies for each pollutant separately only if an abatement option for one pollutant does not affect emissions of others for which reduction targets exist, and if application of an abatement technology for one pollutant does not affect the applicability of abatement technologies for others. An abatement option for one pollutant, however, may affect emissions of other pollutants, and application of an abatement technology for one pollutant may exclude abatement technologies for others. Consequently, cost-effective strategies for simultaneous emission reductions of several pollutants can only be determined in an integrated analysis.

In the model, abatement options are no longer attributed to a specific pollutant. An abatement option now may have impacts on various pollutants, either reducing or increasing emissions, and reducing emissions of at least one pollutant. When the application of an abatement technology excludes the application of another, the combination of these technologies is not included as an abatement option in set $N$. The parameter $\rho_{k,n,p}$ represents the effect of abatement option $n \in N$ on emissions of pollutant $p \in P$ at source $k$. For $0 < \rho_{k,n,p} \leq 1$, emissions of $p$ decrease (to a maximum of 100%); for $\rho_{k,n,p} = 0$, there is no effect on emissions of $p$; and for $\rho_{k,n,p} < 0$, emissions of $p$ increase (possibly more than 100%).

With several pollutants included in the model, Eqs. 10 and 11 replace Eqs. 3 and 4 (other equations remain unchanged):

$$e_{k,p} = \varepsilon_{k,p} \cdot \bar{X}_k \cdot \left(1 - \sum_{n \in N} a_{k,n} \cdot \rho_{k,n,p}\right) \tag{10}$$

$$\sum_{k \in K} e_{k,p} \leq \bar{E}_p \quad \forall p \in P \tag{11}$$

Cost-effective abatement strategies to achieve the emission targets for all pollutants cannot be determined by ranking the abatement options according to their marginal costs. The marginal cost approach as described earlier can only be applied if emissions of all pollutants can be added up (e.g., using weighting factors for individual pollutants) and a tradeoff is allowed between abatement of various pollutants (i.e., emission increases of one pollutant compensated by emission reductions of another). This is possible if all pollutants contribute to the same environmental effect (e.g., acid equivalents for acidifying compounds, global warming potentials for greenhouse gases). If several environmental issues are considered, no abatement cost curve can be constructed. To be able to include several environmental problems in the model, cost-effective abatement strategies are determined by numerically solving the optimisation problem for specific emission constraints. Abatement options that are cost-effective for one set of emission constraints may be less cost-effective or not cost-effective at all for another set of constraints, depending on their costs and effects on emissions of various pollutants. Moreover, if an abatement option causes an increase in the emissions of a pollutant, the cost-effectiveness of this option also depends on the availability and the cost of other abatement options to reduce the emissions of this pollutant.

The model was used to analyse cost-effectiveness of strategies to reduce emissions of ammonia ($NH_3$), nitrous oxide ($N_2O$), and methane ($CH_4$) from European agriculture, considering interactions between abatements of these gases. Emissions of these pollutants (included in set *P*) are to a large extent associated with the same agricultural activities. The model distinguishes 36 countries and in each country 14 categories of activities: 11 livestock categories, synthetic fertiliser use (2 types), and nitrogen fertiliser production, resulting in more than 500 emission sources (set *K*). Activity levels were taken from databases in the RAINS model[x], which includes data for 1990, 1995, and projections up to 2010[6]. Emission factors for $NH_3$ were taken from the RAINS model. Emission factors for $N_2O$ and $CH_4$ were determined according to the method described by Brink et al.[19].

Abatement options in the model (set *N*) include abatement technologies for $NH_3$, $N_2O$, and $CH_4$, and possible combinations of up to six of these technologies, which results in about 750 abatement options in total[xi]. Information on technologies to reduce $NH_3$ was taken from the RAINS model[21]. For technologies particularly aiming at reducing emissions of $N_2O$ and $CH_4$ from agriculture, information was taken from Hendriks et al.[22] and Bates[23,24,25]. Details about these abatement options, their costs, and effects on emissions are given in Brink[26]. In Brink et al.[4] several scenarios have been presented.

## ENVIRONMENTAL POLICY INSTRUMENTS

For environmental protection, the government basically has the options to apply the following policy instruments:

- Direct regulation

- Economic instruments:
  - Taxes, charges
  - Subsidies
  - Tradable permits
  - Deposit systems
- Voluntary agreements
- Government provision of environmental services
- Information, education, and propaganda

In practice, we observe a wide variety of environmental policy instruments within Europe and the U.S. and in the area of international environmental agreements. To select the most appropriate policy instrument, it is essential to have a detailed understanding of the characteristics of the environmental problem in question, of the technological options to reduce pollution, and of the complications that may occur in the application of the instrument.

A very important distinction for the choice of policy instruments is between *uniformly* mixing pollutants and *nonuniformly* mixing pollutants. Uniformly mixing pollutants are dispersed evenly in the atmosphere, like carbon dioxide. For these it does not really matter at which location the emissions occur: the result for the global environment will be the same. Nonuniformly mixing pollutants, however, have a regionally specific impact: the concentrations are higher closer to the point of emissions.

Tradable permits are very well-suited for uniformly mixing pollutants, because *the location* of the emission is irrelevant. For nonuniformly mixing pollutants, it is recommended to use region- or site-specific instruments, like specific standards or differentiated environmental charges that are specific for various locations and regions.

It is extremely complicated to introduce systems of tradable permits, although the experience so far is rather positive, because emission reduction can be obtained at relatively low costs. For uniformly mixing pollutants, a system of tradable permits at the global level would be appropriate. For nonuniformly mixing pollutants, trade should be restricted to certain regions in order to avoid accumulation of emissions at certain locations.

It is important to note that a well-designed system of standards can also be very efficient, provided that it takes into account differences in abatement costs for various producers. If all information on emission-reduction costs is available, a social planner could calculate the cost-efficient solution and could act accordingly in setting emissions standards for individual firms. In practice, this information is not available or is only partially available, and that is the reason that direct regulation often leads to inefficient solutions.

Incentives for technological progress, however, are best provided by economic instruments: tradable emission permits or systems of emission charges are very effective to reduce emissions, provided that the systems of collection of charges is reliable and that monitoring and enforcement can be effectively implemented. These systems continue to provide incentives for producers to search for low-cost, cleaner technologies, because these will reduce the number of permits that the firm has to purchase or reduce the emission charges that have to be paid.

Tradable emission permits and emission charges for various pollutants provide the correct incentives to reduce several pollutants simultaneously, because both systems provide correct incentives to chose those technologies that contribute to emission reduction in the most efficient manner. For example, if charges for carbon dioxide emissions and for acidifying emissions are implemented, producers will chose those technologies that reduce both types of pollutants at the same time, and they will continue to search for technologies that enable them to pay less charges to the government.

International environmental agreements are needed to solve transboundary problems efficiently. Usually the international agreements focus on establishing the emission ceilings for the participating countries, like in the Gothenburg and the Kyoto Protocol. Next, the parties in the Protocol have to implement their policies to reduce emissions to the agreed level. For cost-effectiveness, it is essential that countries chose those policy instruments that allow for flexibility, that provide incentives for technological innovations, and that allow for cost-efficient solutions if multipollutants and joint emission-reduction options are at stake.

Because all policy instruments have impacts on the distribution of costs and benefits over countries, strong incentives exist for free-riding. The allocation of tradable permits over various countries or the redistribution of tax revenues could be used to provide incentives to join the coalition and to reduce the incentives for free-riding. Other reasons to join the coalition could be international solidarity or political pressure on countries that otherwise would not be willing to participate.

## CONCLUSIONS

In this paper, we have studied interrelations between policies for various environmental problems and several policy instruments to reduce emissions. Interrelations may exist when (1) one source emits several pollutants contributing to different environmental problems, (2) abatement of one pollutant also affects other emissions (either beneficially or adversely), (3) one pollutant contributes to several environmental problems, and (4) one environmental problem influences other environmental effects. These interrelations may largely affect cost-effectiveness of abatement strategies but are usually neglected in designing environmental policies.

We present a comparative static optimisation model to analyse cost-effectiveness of environmental policies with respect to various environmental problems, taking into account interrelations between these policies. A large number of technical measures to reduce emissions can be considered, taking into account their costs and effects on emissions of pollutants included. The model applies a stepwise linear approach to calculate emissions, emission reductions, and abatement costs and minimises total cost to meet specified emission-reduction targets.

In a separate paper[4], we applied the model to the agricultural sector in Europe, which is an important source of $NH_3$ (contributing to acidification and eutrophication) and of the greenhouse gases $N_2O$ and $CH_4$. The model was used to estimate these emissions from agriculture in 36 European countries in 1990 and 2010. For 2010, we compared various scenarios assuming different restrictions on emission levels and determined cost-effective strategies to meet these targets. Several technical measures to reduce emissions from agriculture were considered, including estimates of the effects on emissions of all three gases.

Our study shows that important interrelations exist in agriculture between emission-reduction strategies for $NH_3$ and for the greenhouse gases $N_2O$ and $CH_4$. Interrelations may also occur in other economic sectors. Increasing greenhouse gas emissions as a result of policies in other areas should not be ignored, because they will make it more difficult and costly to meet the reduction targets for greenhouse gas emissions. Considering commitments of (in particular western) European countries to reduce emissions of these gases, considerable cost savings can be obtained if side effects of emission control options are explicitly included in the policy-making process.

In interpreting the results of the study, one should be cautious, because there are uncertainties on the exact shape of the costs of the various emission-reduction technologies, on the emission factors used in the model, and on the specification of the interactions in the various abatement technologies. We are, however, convinced that the main line of analysis holds despite these uncertainties.

The choice of policy instruments is extremely important for providing incentives for the design and implementation of clean technologies to reduce emissions and for implementing cost-efficient solutions. Economic instruments, like tradable emission permits or pollution charges, are most suited to providing these incentives, because they imply direct cost savings for firms if they reduce emissions. However, in the design of systems that are based on economic instruments, it is important to make a distinction between *uniformly* mixing pollutants and *nonuniformly* mixing pollutants. For the latter category of problems, economic instruments are more difficult to apply.

For those cases where interactions between pollutants and joint emission reductions are available, economic instruments are to be preferred over other instruments, because economic instruments provide correct incentives for choosing technologies that simultaneously reduce various pollutants.

It is an interesting question whether the international environmental agreements (on climate change or on other topics of transboundary environmental problems) will be stable and will provide incentives to individual countries to join the agreement. The Kyoto Protocol is an interesting test case to see if and when countries that are not yet participating will join the agreement.

## ACKNOWLEDGEMENTS

Paper presented at the International Conference on Environmental Concerns and Emerging Abatement Technologies, Beijing, China, October 9–12, 2001. This project was financed by NWO in the context of the IIASA/SENSE cooperation. The authors would like to thank Z. Klimont and M. Amann at the Transboundary Air Pollution Project at IIASA, Laxenburg, Austria for their assistance in this project.

## ENDNOTES

[i] Regional Air pollution INformation and Simulation (RAINS) model, developed at the International Institute for Applied Systems Analysis (IIASA) as a tool for the integrated assessment of alternative strategies to reduce air pollution in Europe[6,27].

[ii] In this paper, the term “air pollutant” is restricted to pollutants contributing to transboundary air pollution problems such as high concentrations of ground-level ozone, acidification, and eutrophication. Greenhouse gases are not included in this term.

[iii] Emissions cannot be reduced by changes in production level or mix of inputs, because activity levels in each sector are exogenous. So far, it was also assumed that activity level $\overline{X}_k$ does not change as a result of applying abatement options.

[iv] If, for example, at source *k* two abatement options are applied at rates $a_{k,1} = 0.6$ and $a_{k,2} = 0.4$, this means that option 1 is applied to 60% and option 2 to the remaining 40% of activities within source *k*.

[v] This means that economies of scale are disregarded.

[vi] The methodology described here to construct the abatement cost curves was taken from the RAINS model[28].

[vii] For $d(k) = 1$, $\mu_{k,d(k)} = \xi_{k,d(k)}$.

[viii] For a gradually increasing emission reduction, there is a smooth transition from option at position $d(k)$ to option position $d(k) + 1$. The application rate for abatement option at position $d(k) + 1$, $a_{k,d(k)+1}$, increases from zero to one, while at the same time $a_{k,d(k)}$ decreases from one to zero, under the restriction that $a_{k,d(k)} + a_{k,d(k)+1} = 1$.

[ix] For $d(k) = 1$, $r_{k,d(k)} = X_k \cdot \varepsilon_k \cdot \rho_{k,d(k)}$.

[x] The agricultural sector was included in the RAINS model to estimate $NH_3$ emissions and to analyse cost-effectiveness of $NH_3$ abatement[29].

[xi] Although abatement options are not attributed to a specific pollutant, they are presented in the literature as options to reduce emissions of a specific pollutant.

## APPENDIX: DETERMINATION OF PARAMETERS FOR COMBINATIONS OF ABATEMENT TECHNOLOGIES

The model contains a set $N$ of abatement options, which includes both single abatement technologies and possible combinations of these technologies. Set $Y$ is defined as the set of single abatement technologies, which is a subset of set $N$. Possible combinations of technologies are included as separate options with their own parameters for cost and effect, calculated on the basis of parameters of the single technologies in the combination. This describes how parameters for combinations can be determined.

Although it is not made explicit in the model, in fact each source $k$ is considered to consist of a number of firms in which similar economic activities take place. The sum of the activity levels in the individual firms gives the activity level for the source:

$$\sum_{f_k \in F_k} \bar{x}_{f_k} = \bar{X}_k$$

with $F_k$ the set of firms $f_k$ that together form source $k$ and $\bar{x}_{f_k}$ the activity level at firm $f_k$. The application rate $a_{k,n}$ represents the fraction of economic activity at source $k$ to which abatement option $n$ is applied. At firm level, abatement options are either fully applied or not applied, i.e., $b_{f,k,n} \in \{0,1\}$, with $b_{f,k,n}$ the application of abatement option $n$ at firm $f_k$. The application rate at source level is

$$a_{k,n} = \frac{\sum_{f_k \in F_k} b_{f_k,n} \cdot \bar{x}_{f_k}}{\bar{X}_k}$$

At firm level, several single abatement technologies $y \in Y$ can be applied together. The joint effect on emissions of these technologies is not the sum of the effects of single technologies. When two technologies are applied together, the second technology only affects the emissions that remain after the first technology has been applied. Consequently, the joint impact will be lower than the sum of the effects of individual technologies. In fact, the additional effect of the second technology on emissions is equal to the removal efficiency of this technology ($\rho_2$) multiplied by the fraction of emissions remaining after application of the first technology ($1 - \rho_1$). Hence, the total impact of the combination of the two technologies is $\rho_1 + \rho_2 - \rho_1\rho_2$. In general, the relative reduction of a combination of technologies is

$$\rho_{k,n} = 1 - \prod_{y \in S(n)} \left(1 - \rho_{k,y}\right) \quad \forall\, n \in N$$

$S(n) \subseteq Y$ is the set of single abatement technologies included in abatement option $n$, and $\rho_{k,y}$ is the effect on emissions of the single technology $y$. The effect on emissions is independent of the order of introduction of the different technologies. The cost per unit of activity of a combination of technologies is equal to the sum of the costs of the single technologies applied:

$$\gamma_{k,n} = \sum_{y \in S(n)} \gamma_{k,y} \quad \forall\, n \in N$$

with $\gamma_{k,y}$ the unit cost of single technology $y$. Once the parameters $\rho_{k,n}$ and $\gamma_{k,n}$ are determined for all possible combinations, they can be treated as single technologies for which the application rate at each source $a_{k,n}$ is determined.

Please note that it would also have been possible to include combinations of technologies directly in the model, but this would result in nonlinearities and even nonconvexities. Consequently, optimisation would result in local optima, and it would be difficult to find the optimal solution. By treating combinations as separate options, this is avoided and the model remains linear.

## BIOSKETCH

**Ekko van Ierland** is Professor of Environmental Economics and Natural Resources at Wageningen University, The Netherlands. He completed his studies and his PhD at the Faculty of Economics at the University of Amsterdam. His research focus is on economics of transboundary air pollution, management of biodiversity, and environmental economic modelling.
